# Association between B Cell Growth Factors and Primary Sjögren's Syndrome-Related Autoantibodies in Patients with Non-Hodgkin's Lymphoma

**DOI:** 10.1155/2019/7627384

**Published:** 2019-01-13

**Authors:** Zhenhua Xian, Dehua Fu, Shuang Liu, Yang Yao, Chun Gao

**Affiliations:** Department of Gastrointestinal Surgery, Tongji Hospital of Tongji Medical College of Huazhong University of Science and Technology, Wuhan 430030, China

## Abstract

Despite the overall success of using R-CHOP for the care for non-Hodgkin's lymphoma patients, it is clear that the disease is quite complex and new insight is needed to further stratify the patient for a better personized treatment. In current study, based on previous studies from animal model, new panels combining well-established cytokine (BAFF) and autoantibodies (anti-SSA/Ro) with newly identified cytokine (IL14) and autoantibodies (TSA) were used to evaluate the association between B cell growth factor and Sjögren's related autoantibodies in NHL patients. The result clearly indicates that there was a unique difference between BAFF and IL14 in association with autoantibodies. While serum BAFF was negatively associated with the presence of both traditional anti-SSA/Ro and novel TSA antibodies in GI lymphoma patient, IL14 was positively associated with the presence of both traditional anti-SSA/Ro and novel TSA antibodies in non-GI lymphoma patient. Long-term follow-ups on these patients and evaluation of their response to the R-CHOP treatment and recurrence rate will be very interesting. Our result provides a solid evidence to support using novel diagnostic panel to better stratify the NHL patients.

## 1. Introduction

Sjögren's syndrome (SS) is a chronic autoimmune disease characterized by lymphocytic infiltrates of salivary and lacrimal glands, leading to oral and ocular dryness and increase of autoantibody secretion [1]. The term primary Sjögren's syndrome (pSS) was used to define SS without the presence of other systemic autoimmune diseases. Occurrence of lymphoma is one of the most severe complications of pSS; about 5% of patients with pSS will develop non-Hodgkin lymphomas (NHL) [2–4], with an estimated risk up to 44 times greater than normal population [5]. Investigation of the pathophysiology of lymphomagenesis associated with the development of pSS will provide a new insight for a better knowledge of the underlying mechanisms for autoimmunity and lymphomagenesis in general, which might lead to the identification of novel treatment targets for autoimmune diseases and lymphoma.

B cells play a central role to the pathogenesis of primary SS, which is characterized by polyclonal B cell hyperactivity, and later switch on monoclonal B cell expansion that results in the development of B cell lymphoma in pSS patients. The correlation of B cell growth factors with pSS has been well established. BAFF (also termed TNFSF13B) promotes B cell maturation, proliferation, and survival. BAFF transgenic mice develop features of SLE and later clinical characteristics of pSS, such as sialadenitis; 3% of these mice develop lymphoma when aged [6]. Recent studies have also demonstrated that BAFF might also be involved in the occurrence of lymphoma in pSS patients [7].

The presence of autoantibodies is one of the several hallmarks of Sjögren's syndrome; the detection of serum autoantibodies has a central role in the diagnosis and classification of Sjögren's syndrome [8]. Anti-SSA/Ro is the most common autoantibodies found in patients with pSS that directed against the autoantigens Ro/La ribonucleoprotein complex. Previous study has demonstrated that serum BAFF levels were enhanced and correlated with levels of anti-SSA/Ro, anti-SSB/La, and RF in pSS patients [9]. Clinical trial based on the monoclonal antibody target BAFF is currently underway to evaluate the efficacy of blocking BAFF for the treatment of pSS and lymphoma.

Recently, another B cell growth factor has been shown to play an important role in the pathophysiology of pSS. IL14, also known as taxilin, was initially identified as a high molecular weight B cell growth factor which can promote B cell proliferation, especially of B cells within the GC [10]. IL14*α* transgenic mice present both clinical and biological characteristics of pSS [11]. When aged, 95% of IL14*α* transgenic mice develop B cell lymphomas in the gastrointestinal tract with histological features of a large B cell lymphoma. Novel tissue-specific autoantibodies (TSA), which include antisalivary gland protein 1 (SP1), anticarbonic anhydrase 6 (CA6), and antiparotid secretory protein autoantibodies (PSP), were first identified from IL14*α* transgenic mouse (IL14*α*TG) and have been found in patients with SS both together and without anti-SSA/Ro, as well as in patients with idiopathic dry mouth and dry eye disease [12]. It was proposed that TSA antibodies may be useful for identifying early SS, particularly among patients who are anti-SSA/Ro negative. While studies on IL14*α* transgenic mice model have led to interest insight about the development of pSS and lymphoma, no clinical studies has been done to evaluate the associations between IL14 and novel autoantibodies in the context of autoimmunity and lymphomagenesis.

In current study we will for the first time investigate the associations between B cell growth factors and primary Sjögren's syndrome-related autoantibodies in patients with non-Hodgkin's lymphoma.

## 2. Materials and Methods

### 2.1. Patients

A total of 139 patients with NHL were enrolled from the Department of Hematology and Department of Gastrointestinal Surgery, Tongji Hospital, Tongji Medical College, Huazhong University of Science and Technology, Wuhan. These patients were diagnosed in accordance with WHO classification and mainly on the basis of histopathological and immunohistochemical findings of biopsy samples. Also included were 8 healthy volunteers, with informed consent obtained in writing. Sera were obtained and tested after informed consent from 118 NHL patients. Peripheral blood leukocytes were collected from the rest of 21 NHL patients and 8 health controls. The study protocol was approved by Tongji Medical College IRB committee.

### 2.2. Western Blot Assays

Western blot assays were run following the manufacturer's instruction. In brief, a total of 4 *μ*l of diluted serum (1 : 100 dilution with PBS) were mixed with 20 *μ*l PBS and 6 *μ*l 5 × loading buffer (Thermo Fisher, Carlsbad, CA). Samples were denatured at boiled water for 8 minutes before loading to 10% dodecyl sulfate-polyacrylamide gel electrophoresis (SDS-PAGE) and then transferred onto a nitrocellulose membrane. The membrane was incubated with blocking buffer (skim milk) for 2 h at room temperature and incubated overnight at 4°C with 1 : 1000 primary antibodies in blocking buffer. After washed three times for 10 min each with TBST (0.02% Tween in TBS) (TBS; 20 mM Tris-HCl, pH 7.4, and 150 mM NaCl), the membrane was incubated with the horseradish peroxidase-labeled goat anti-mouse IgG secondary antibody (diluted 1 : 5000 in blocking buffer) for 2 h at room temperature. The membrane was washed three times for 10 min each with TBST before Pierce ECL Western blotting substrate kit containing goat anti-rabbit IgG conjugate with horseradish peroxidase (Thermo Scientific, Waltham, MA) which was used to visualized the result in a Machine (Bio-Rad, Hercules, CA).

### 2.3. Determine the Relative Intensity Ratio for Serum IL14*α* Level after WB Assay

For comparison purpose, an internal positive control was used throughout the whole study for normalization purpose between different batches of gels. Based on internal control, a ratio of mean density value can be calculated to reflect the actual relative expression level of IL14*α*.

### 2.4. ELISA

For serum BAFF level, ELISA was run following the manufacturer's instruction (R&D Systems, Minneapolis, MN). For Sjögren's related autoantibodies (Ro, La, SP1, PSP, and CA6), ELISA kits were purchased from Trinity Biotech, Buffalo, NY.

### 2.5. Quantitative PCR

Fresh blood was obtained from patients with NHL and normal control. Total RNAs are isolated from the blood using QIAamp RNA Blood Mini kit (Qiagen) following the manufacturer's instructions. cDNA was produced from total RNA using the SuperScript first-strand synthesis system for RT-PCR according to the manufacturer's instructions (Thermo Fisher, Carlsbad, CA). Quantitative PCR (qPCR) reaction was set up using SYBR Select Master Mix purchased from Thermo Fisher (Carlsbad, CA) following the manufacturer's instructions. qPCR was run on an StepOnePlus real-time PCR system (Life Technologies, Carlsbad, CA) with the following program: 95°C for 30 s hold, 40 cycles of 95°C, 5 s and 60°C, 31 s. Melting curve analysis was performed: 95°C, 15 s, 60°C, 60 s, 95°C, 15 s. The primers for IL14 were forward primer 5′-TCACAGAAGCGCCTTGCTA-3′ and reverse primer 5′-CCAGTCTGGCCTGATGCTT-3′, and for 18S rRNA control were forward primer 5′-CGCGGTTCTATT TTGTTGGT-3′ and reverse primer 5′-AGTCGGCATCGTTTATGGTC-3′.

### 2.6. Statistical Analysis

Data were statistically analyzed with SPSS (version 16.0) or Prism (version 6.0, GraphPad software). Data are presented as mean ± standard deviation (SD). To compare the difference between two groups, unpaired two-tailed Student′s test was performed. Pearson correlation coefficient was used to analyze the correlations between two variables. *P* values < 0.05 were considered statistically significant.

## 3. Results

### 3.1. Expression of IL14*α* Gene in Peripheral Blood Leukocytes of Lymphoma Patients

First, peripheral blood leukocytes were collected from 3 groups of patients (Table 1), and real-time PCR assay was run to evaluate the expression of IL14*α* gene in the peripheral blood leukocytes of lymphoma patients compared to healthy control.

**Figure 1:**
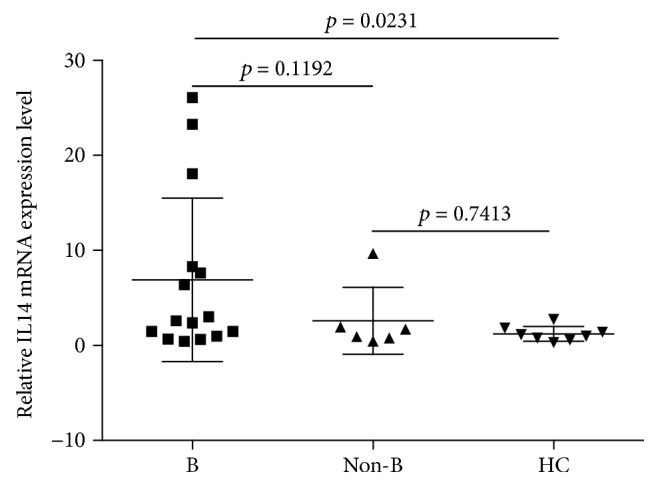
IL14*α* expression of IL14*α* in peripheral blood leukocytes of B cell lymphoma patients, non-B cell lymphoma patients, and HC.

As shown in Figure 1, IL14*α* level in peripheral blood leukocytes of HC group was 1.21 ± 0.78, B cell lymphoma group was 6.89 ± 8.59 (*p* = 0.0231), and non-B cell lymphoma group was 2.57 ± 3.52 (*p* = 0.7413). IL14*α* level in peripheral blood leukocytes of B cell lymphoma group significantly increased compared to HC group (*p* = 0.0231). IL14*α* level in B cell lymphoma group also increased compared to non-B cell lymphoma group, but with no significant statistical difference (*p* = 0.1192).

### 3.2. Patient Population and Their Basic Clinical Characteristics

Next, serum from 118 NHL patients was collected to evaluate the associations between B cell growth factors and Sjögren's related autoantibodies. For analysis purpose, these NHL patients were divided into two groups using two different methods, respectively. The first method was based on the location of focus: 26 patients with gastrointestinal (GI) lymphoma, 92 patients with nongastrointestinal (non-GI) lymphoma (Table 2). The other method was based on pathologic phenotypes: 86 patients with B cell lymphoma and 32 patients with non-B cell lymphoma (Table 3). Sera were obtained and basic clinical characteristics were collected from these people to evaluate the expression of BAFF, IL14, and Sjögren's related autoantibodies.

### 3.3. Serum IL14*α* and BAFF Levels in GI Lymphoma, Non-GI Lymphoma, B Cell Lymphoma, and Non-B Cell Lymphoma Groups

**Figure 2:**
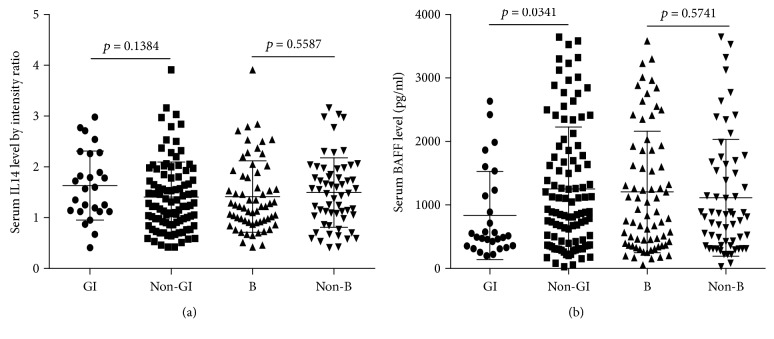
(a) Relative intensity ratio for serum IL14*α* level in GI lymphoma, non-GI lymphoma, B cell lymphoma, and non-B cell lymphoma groups. (b) Serum BAFF level in GI lymphoma, non-GI lymphoma, B cell lymphoma, and non-B cell lymphoma groups.

The relative intensity ratio for serum IL14*α* level in GI lymphoma group was 1.63 ± 0.68, non-GI lymphoma group was 1.41 ± 0.69, B cell lymphoma group was 1.42 ± 0.70, and non-B cell lymphoma group was 1.49 ± 0.68. Serum BAFF level (pg/ml) in GI lymphoma group was 834.2 ± 694.3, non-GI lymphoma group was 1251 ± 976.6, B cell lymphoma group was 1206 ± 955.9, and non-B cell lymphoma group was 1113 ± 920.6. There was no difference of serum IL14 *α* level between GI lymphoma and non-GI group (*p* = 0.1384) as well as B cell lymphoma and non-B cell lymphoma group (Figure 2(a)). Serum BAFF level in GI lymphoma group significantly increased compared to non-GI lymphoma group, while B cell lymphoma had no difference with non-B cell lymphoma (Figure 2(b)).

### 3.4. The Association among Serum IL14*α* Level, Serum BAFF Level, and Autoantibodies in Lymphoma Patients

**Figure 3:**
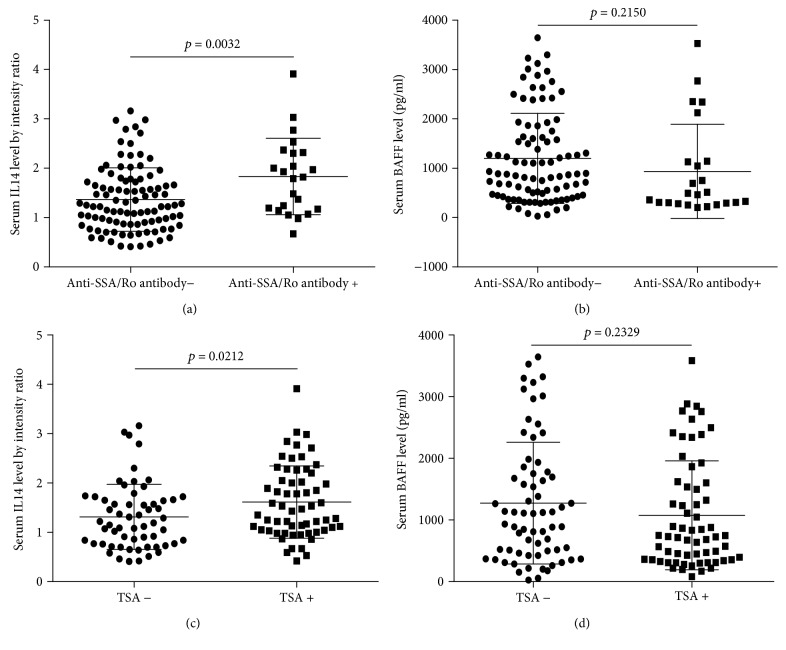
(a) The association between serum IL14*α* level and anti-SSA/Ro antibody in lymphoma patients. (b) The association between serum BAFF level and anti-SSA/Ro antibody in lymphoma patients. (c) The association between serum IL14*α* level and TSA in lymphoma patients. (d) The association between serum BAFF level and TSA in lymphoma patients.

It was shown that serum IL14*α* level in anti-SSA/Ro antibody positive group and TSA positive group was significantly increased compared to anti-SSA/Ro antibody negative group (*p* = 0.0032) and TSA negative group (*p* = 0.0212), while BAFF did not show a significant difference (*p* = 0.2150) (*p* = 0.2329) (Figures 3(a)–3(d)).

### 3.5. The Association among Serum IL14*α* Level, Serum BAFF Level, and Autoantibodies in GI Lymphoma and Non-GI Lymphoma Patients

**Figure 4:**
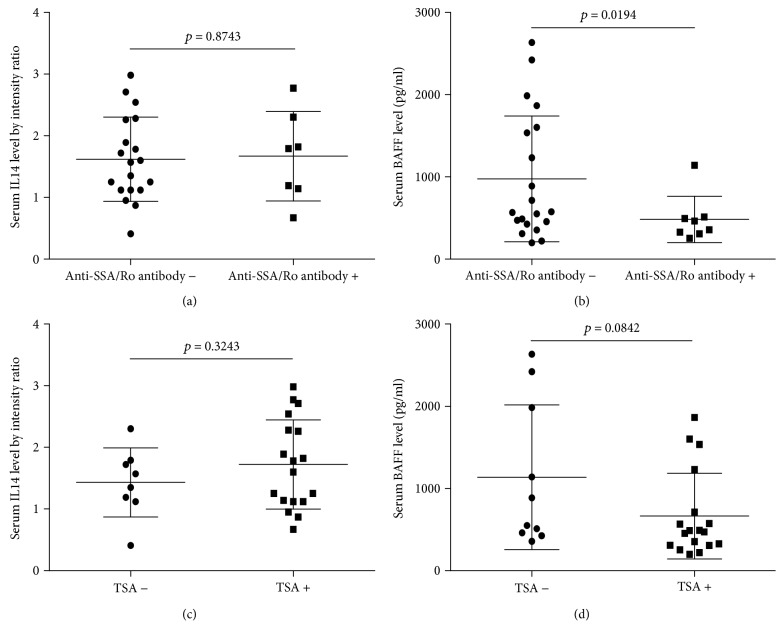
(a) The association between serum IL14*α* level and anti-SSA/Ro antibody in GI lymphoma patients. (b) The association between serum BAFF level and anti-SSA/Ro antibody in GI lymphoma patients. (c) The association between serum IL14*α* level and TSA in GI lymphoma patients. (d) The association between serum BAFF level and TSA in GI lymphoma patients.

**Figure 5:**
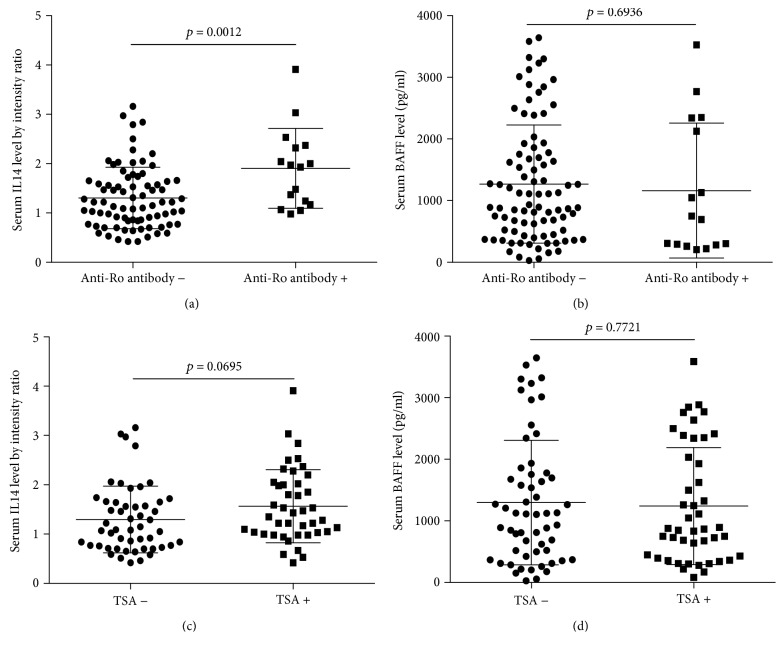
(a) The association between serum IL14*α* level and anti-SSA/Ro antibody in non-GI lymphoma patients. (b) The association between serum BAFF level and anti-SSA/Ro antibody in non-GI lymphoma patients. (c) The association between serum IL14*α* level and TSA in non-GI lymphoma patients. (d) The association between serum BAFF level and TSA in non-GI lymphoma patients.

In GI lymphoma patients, we found that there is no difference with serum IL14*α* level between autoantibodies negative and positive patients (Figures 4(a) and 4(c)); serum BAFF level was associated with the classical autoantibodies anti-SSA/Ro not TSA (Figures 4(b) and 4(d)). In non-GI lymphoma patients, the results showed serum IL14*α* level was associated with the classical autoantibodies anti-SSA/Ro, but it was not associated with TSA (Figures 5(a) and 5(c)). Whereas the serum level of BAFF (pg/ml) in non-GI lymphoma had no association with autoantibodies (Figures 5(b) and 5(d)).

## 4. Discussion

Despite the overall success of R-CHOP, the standard of care for non-Hodgkin's lymphoma, it is clear that many patient subsets are not cured. Based on the recognition of major genetic and biologic subtypes harboring distinct pathogenetic lesions, our understanding of non-Hodgkin's lymphoma biology evolves; it is clear that the disease is quite complex and new insight is needed to further stratify the patient for a better personized treatment.

Patients that develop autoimmune syndromes such as SLE, Sjögren's syndrome, and RA have an increased risk of developing B cell malignancies [13], which suggests there may be underlying common mechanisms between the development of both autoimmunity and lymphoid malignancies. A good example will be that excessive production of BAFF is not only associated with the development of a range of mature B cell malignancies but also play an important role in the development of pSS, indicating that BAFF may be an important molecular link between autoimmunity and cancer.

In pSS, BAFF expression is elevated and acts as a link between innate immune activation and chronic autoimmune B cell activation. Overactivation of B cells in patients is associated with the higher frequency of non-Hodgkin's B cell lymphomas found in pSS patients compared with the general population [14]. It is well established that BAFF is critical for B cell survival in the periphery, and abundant BAFF expression also contributes to the reduced levels of B cell apoptosis in SS salivary gland cells and subsequent excessive B cell activation and increased risk of lymphoma. It was also demonstrated that serum BAFF levels were enhanced and correlated with levels of autoantibodies such as anti-SSA/Ro, anti-SSB/La, and RF in pSS patients [9].

Recently, another B cell growth factor, IL14, has been shown to also play an important role in the development of pSS- and SS-associated lymphoma. It was hypothesized that IL14 can selectively act on memory B cell to enhance memory B cell function and induces SS by converting low-affinity autoreactivity into high-affinity memory B cell [8, 15, 16], while both IL14 and BAFF transgenic mice shared lots of similar features as animal model for pSS, such as lymphocytic infiltration of lacrimal and submandibular gland. In the absence of MZB cell, both animal models did not develop SS phenotypes [17, 18]. There was a significant difference between this two animal models such as BAFF transgenic mice that do not spontaneously develop lymphoma, as in IL14*α*TG mice, and they develop more severe proliferative glomerulonephritis [19].

In current study, based on previous studies from animal model, new panels combining well-established cytokine (BAFF) and autoantibodies (anti-SSA/Ro) with newly identified cytokine (IL14) and autoantibodies (TSA) were used to evaluate the association between B cell growth factor and Sjögren's related autoantibodies in NHL patients. The result clearly indicated that there was a unique difference between BAFF and IL14 in association with autoantibodies. While serum BAFF was negatively associated with the presence of both traditional anti-SSA/Ro and novel TSA antibodies in GI lymphoma patient, IL14 was positively associated with the presence of both traditional anti-SSA/Ro and novel TSA antibodies in non-GI lymphoma patient. Long-term follow-ups on these patients and evaluation of their response to the R-CHOP treatment and recurrence rate will be very interesting. Our result provided a solid evidence to support using novel diagnostic panel to better stratify the NHL patients.

Sjögren's syndrome is one of the most common autoimmune disease in adults; however, SS is greatly under recognized in clinical practice, mostly due to diverse symptomatic expressions making the initial diagnosis difficult. It is estimated that the disease remains undiagnosed in more than half of affected adults [20, 21]. While previous long-term follow-up studies have shown about 5% of patients with pSS will develop non-Hodgkin lymphomas (NHL) later as the disease progresses, there is no study to evaluate how many NHL patients may have an underlying Sjögren's syndrome. Since all NHL patients in current study were enrolled from the Department of Hematology and Department of Gastrointestinal Surgery, there was no clinical assessment to evaluate whether these patients meet the diagnostic criteria of SS (usually done by rheumatologist). To our surprise, as shown in current study, there were significant high number of NHL patients positive for SS-associated autoantibodies: about 20% (23 out of 118) for anti-SSA autoantibodies and 50% (59 out of 118) for TSA autoantibodies. For future clinical studies, it will be very interesting to gather additional clinical information (such as dry eye/dry mouth evaluation and minor salivary gland biopsy) for those SS-associated autoantibodies positive NHL patients to fully evaluate the potential clinical significance to stratify the NHL patients for better personized treatment.

Due to the availability of limited clinical information, we were also not able to run in-depth analysis in current study to address question such as what is the difference in the clinical manifestations and prognosis between these SS-associated antibody positive NHL patients and the others. This will be addressed in our future studies based on the interesting finding in our current study.

## Figures and Tables

**Table 1:** Basic clinical characteristics of study groups.

Group	Total case number	Sex (male/female)	Age
B cell lymphoma	15	11/4	39.92 ± 17.70
Non-B cell lymphoma	6	4/2	45.00 ± 10.63
Healthy control	8	6/2	27.25 ± 3.37

**Table 2:** Basic clinical characteristics of study groups (basic on location of focus).

Group	Total case number	Sex (male/female)	Age
Gastrointestinal (GI) lymphoma	26	14/12	48.77 ± 15.75
Nongastrointestinal (non-GI) lymphoma	92	55/37	46.95 ± 16.57

**Table 3:** Basic clinical characteristics of study groups (basic on pathologic types).

Group	Total case number	Sex (male/female)	Age
B cell lymphoma	86	47/39	50.26 ± 14.88
Non-B cell lymphoma	32	22/10	39.53 ± 17.73

## Data Availability

The original data used to support the findings of this study are available from the corresponding author upon request.
